# Nurse infected with Covid-19 from a provisional dengue patient

**DOI:** 10.1080/22221751.2020.1775131

**Published:** 2020-06-15

**Authors:** Wisit Prasitsirikul, Krit Pongpirul, Wannarat A. Pongpirul, Nayot Panitantum, Anuttra C. Ratnarathon, Thiravat Hemachudha

**Affiliations:** a Department of Disease Control, Bamrasnaradura Infectious Diseases Institute, Nonthaburi, Thailand; b Department of Preventive and Social Medicine, Faculty of Medicine, Chulalongkorn University, Bangkok, Thailand; c Department of International Health, Johns Hopkins Bloomberg School of Public Health, Baltimore, MD, USA; d Thai Red Cross Emerging Infectious Diseases Health Science Centre, World Health Organization Collaborating Centre for Research and Training on Viral Zoonoses, King Chulalongkorn Memorial Hospital, Faculty of Medicine, Chulalongkorn University, Bangkok, Thailand

**Keywords:** Covid-19, SARS-CoV-2, dengue, nosocomial infection, personal protective equipment

## Abstract

We report a 35-year-old female nurse who possibly received the SARS-CoV-2 virus during the blood sampling of a 35-year-old male patient initially suspected as a dengue infection. The patient had mild thrombocytopenia and positive dengue IgG and IgM whereas the clinicians were not aware of the possibility of false-positive dengue serology revealed in the published case report from Singapore. The nurse put on a pair of gloves but did not wear a mask during the only encounter with this patient. This nosocomial transmission raised a safety concern among healthcare professionals in an area with a relatively low Covid-19 prevalence, especially when the clinical and laboratory characteristics could be confused with other viral infections.

Healthcare professionals are at risk of getting an infection while they are providing care to the patients. More than two thousand Chinese healthcare workers had been infected with coronavirus disease 2019 (Covid-19) because of the lack of awareness and incorrect use of personal protective equipment (PPE) [1]. As clinical and laboratory characteristics of Covid-19 are non-specific [2] and could be similar to other viral infections, healthcare workers might be “distracted” by a provisional diagnosis of dengue infection that is locally common and, therefore, did not have appropriate infection control practice. We report the first Covid-19 infection in a nurse locally transmitted from a patient initially suspected dengue infection during blood sampling.

On January 25, a 35-year-old salesman at a local shop approximately 15 kilometers from Suvarnabhumi airport developed fever, myalgia, mild productive cough, nausea, and vomiting. He usually wears a mask while selling shoes. He had no history of travel to China, but he had some encounters with Chinese tourists at the shop. On January 28, his symptoms were not improved so he decided to get medical care at a local private hospital covered by his Social Security Scheme. The physician prescribed some medications for his symptoms and asked him to come back for follow-up on the next day. On January 29, he went back for the follow-up visit but the symptoms were not improved.

On January 30, his symptoms became worse, so he decided to go to another private hospital. His body temperature was 37.8 degrees Celsius, pulse rate 88 per minute, blood pressure 114/64 millimeters of mercury, oxygen saturation 99%. Nasopharyngeal swab tested negative for influenza A, influenza B, and respiratory syncytial virus. Dengue infection was suspected, and he was admitted. At the hospital ward, after being informed that dengue infection was suspected, a 35-year-old nurse put on only a pair of gloves but did not wear a mask. The initial blood tests revealed mild thrombocytopenia and positive dengue IgG and IgM. The chest radiograph revealed reticular infiltration. At that time, the clinicians were not aware of the possibility of false-positive dengue serology revealed in a case report from Singapore [3].

The clinical conditions of the patient had been worse during the first three days of admission. On February 2, he reported shortness of breath; the chest radiograph revealed progressive infiltration. As Covid-19 was suspected, throat and nasopharyngeal swabs were collected and tested positive for SARS-CoV-2 on real-time reverse-transcription – polymerase-chain-reaction (RT–PCR).

The nurse who took his blood sample developed a fever on February 4. She had no underlying conditions except the Hemoglobin E trait. She had lived alone in a dormitory and had not been outside for non-work-related business. None of her co-workers or other patients who visited the hospital during the time were reported to have Covid-19 infection at the time. Identified as a close contact of the index case during the blood sample collection, the nurse was isolated at the private hospital. Her throat and nasopharyngeal swabs collected on February 6 tested positive for SARS-CoV-2 on RT–PCR so she was admitted on February 8. She was considered the first Thai healthcare personnel who got Covid-19 infection. She was prescribed with oral lopinavir and ritonavir as well as intravenous ceftriaxone. She reported shortness of breath on February 13; the chest radiograph revealed pneumonia of left lower lung so the medications were changed to darunavir, ritonavir, meropenem, oral chloroquine, and zinc. As her pneumonia rapidly worsened, she was referred to Bamrasnaradura Infectious Diseases Institute (BIDI) on February 15. Oral favipiravir was added and her clinical conditions were improved. The nurse was discharged after her RT–PCR became negative and isolated at home for another 14 days before resuming her work at the private hospital.

All individuals who were in close contact with the nurse and male patients were interviewed and underwent RT–PCR tests but none were positive for Covid-19. Twenty-five hospital staff and 21 patients and relatives who were in close contact with the nurse tested negative for SAR-CoV-2 on RT–PCR. Two of the 25 hospital staff had minor upper respiratory tract symptoms and were asked to rest at home whereas the other 23 were allowed to resume their duty during the two-day waiting period.

While standard infection control measure is essential, the provisional diagnosis of dengue infection reduced the awareness of Covid-19 and, therefore, got the nurse infected. Had the patient been suspected only Covid-19, a more appropriate personal protective equipment (PPE) would have been used. The Ministry of Public Health mandated PPE availability in all hospitals and the PPE was readily available at the private hospital during the incident so compliance was the key problem. The blood drawing at the hospital ward usually takes 5–10 minutes and was the only encounter between the nurse and the patient. As the nurse did not wear a mask, the droplets were the potential route of transmission.

This nosocomial transmission has raised a safety concern among healthcare professionals, especially when the clinical and laboratory characteristics of Covid-19 are non-specific and could be confused with other viral infections.
